# Two additional new species of the *Stenus indubius* group (Coleoptera, Staphylinidae) from China

**DOI:** 10.3897/zookeys.215.3702

**Published:** 2012-08-17

**Authors:** Liang Tang, Li-Zhen Li, Jun-Wang Wang

**Affiliations:** 1 Department of Biology, Shanghai Normal University, 100 Guilin Road, 1st Educational Building 323 Room, Shanghai, 200234 P. R. China; 2 Qingliangfeng Nature Reserve administration, Changhua Town, Lin’an City, Zhejiang, 311321 P. R. China

**Keywords:** Coleoptera, Staphylinidae, *Stenus indubius* group, new species, China

## Abstract

Two new species of the *Stenus indubius* group from China are described: *Stenus huapingensis* **sp. n.** from Guangxi Province and *Stenus zhujianqingi***sp. n.** from Zhejiang Province. Habitus photos and illustrations of diagnostic characters of the new species and two described species, *Stenus paradecens* Tang & Li, 2005 and *Stenus guniujiangense* Tang & Li, 2005, are provided.

## Introduction

After our recently published work (Tang and Li 2012) on Chinese species of the *Stenus indubius* group, we received new material containing two new species of the group collected from Guangxi and Zhejiang provinces, respectively. These new species are described in the present paper. One of them is closely related to *Stenus paradecens* Tang & Li, 2005 and *Stenus guniujiangense* Tang & Li, 2005. However, the figures in the original descriptions of the latter two species have minor flaws and are poorly printed. Therefore, new and improved illustrations are provided.

## Material and methods

The specimens examined in this paper were collected by sifting leaf litters in forests. For examination of the male genitalia, the last three abdominal segments were detached from the body after softening in hot water. The aedeagi, together with other dissected pieces, were mounted in Euparal (Chroma Gesellschaft Schmidt, Koengen, Germany) on plastic slides. Photos of sexual characters were taken with a Canon G7 camera attached to an Olympus SZX 16 stereoscope; habitus photos were taken with a Canon macro photo lens MP-E 65 mm attached to a Canon EOS60D camera.

The type specimens treated in this study are deposited in the following public and private collections:

SHNU Department of Biology, Shanghai Normal University, P. R. China

**cPut** private collection V. Puthz, Schlitz, Germany

The measurements of proportions are abbreviated as follows:

BL body length, measured from the anterior margin of the clypeus to the posterior margin of abdominal tergite X

FL length of forebody, measured from the anterior margin of the clypeus to the apex of the elytra (apico-lateral angle)

HW width of head including eyes

PW width of pronotum

EW width of elytra

PL length of pronotum

EL length of elytra, measured from humeral angle apico-lateral angle

SL length of elytral suture

## Taxonomy

### Stenus huapingensis

Tang, Li & Huang sp. n.

urn:lsid:zoobank.org:act:FFC0FF4A-0EA5-4526-927F-1C3791815B75

http://species-id.net/wiki/Stenus_huapingensis

Figs 1 , 5

#### Type material.

**Holotype** ♂:“China: Guangxi Prov., Lingui County, Huaping N. R., Anjiangping, 1500m, 18.VII.2011, TANG Liang Leg.” “Holotype / *Stenus huapingensis* / Tang & Li” [red handwritten label] (SHNU). **Paratypes.** 5♂♂, 12♀♀, same locality but 1400–1700 m, 14–18 VII.2011, L. Tang, W.-J. He, Z. Peng, Y. Chen & W.-L. Ma leg. (1 pair in cPut, remainder in SHNU).

#### Description.

Brachypterous; body blackish, anterior margin of labrum brownish, antennae, maxillary palpi and legs yellowish brown, each elytron with an oval orange spot near lateral side, which is about 1/3 as long as and 1/3 as broad as the respective elytron.

BL: 4.1–4.9 mm; FL: 2.1–2.3 mm.

HW: 0.84–1.02 mm, PL: 0.71–0.82 mm, PW: 0.62–0.72 mm, EL: 0.73–0.88 mm, EW: 0.71–0.89 mm, SL: 0.57–0.65 mm.

Head 1.12–1.18 times as wide as elytra; interocular area with deep longitudinal furrows, median portion convex, extending to the level of inner eye margins; punctures round, well delimited in median portion and more or less confluent into diagonal stria in furrows, slightly larger and sparser in median area than near inner margins of eyes, diameter of large punctures about as wide as apical cross section of antennal segment II; interstices partially with faint reticulation, smaller than half the diameter of punctures except those in median portion and behind basi-antennal tubercles, which may be much larger. Antennae, when reflexed, extending a little beyond posterior margin of pronotum; relative length of antennal segments from base to apex as 11: 8: 19: 11.5: 10: 9.5: 9.5: 6.5: 7: 8: 8.5. Paraglossa oval.

Pronotum 1.11–1.15 times as long as wide; disc with distinct median longitudinal furrow, two indistinct impressions in anterior half, indistinct transverse impression in the middle, and two indistinct impressions in posterior half; punctures moderately rugose and confluent, a little larger than those of head; interstices partially indistinctly reticulated, mostly smaller than half the diameter of punctures except those at the bottom of median longitudinal furrow, which may be distinctly larger.

Elytra 0.94–1.03 times as long as wide, lateral margins with slight concavity before the middle; disc slightly uneven with shallow longitudinal humeral impression, shallow postero-lateral impression, and distinct sutural impression; suture moderately convex; punctation and interstices similar to those of pronotum.

Hind tarsi 0.73 times as long as hind tibiae, tarsomeres IV distinctly bilobed.

Abdomen cylindrical; distinct paratergites absent, but rudimentary lateral border present; tergite VII with palisade fringe; punctures on abdominal tergites III–VIII round to elliptic, gradually becoming smaller posteriad; interstices smaller to little larger than half the diameter of punctures, with relatively faint reticulation on all abdominal tergites.

Male. Sternite VII with posteromedian portion slightly flattened; sternite VIII (Fig. 5A) with shallow emargination at middle of posterior margin; sternite IX (Fig. 5B) with very long apico-lateral projections, posterior margin serrate; tergite X (Fig. 5C) with posterior margin slightly emarginated. Aedeagus (Figs. 5D, 5E) with setae at sclerotized apex of median lobe; expulsion hooks (Fig. 5G) large; parameres extending beyond apex of median lobe, bisinuate, with about 13 setae on inner side (Fig. 5F).

Female. Abdomen broader than in male; sternite VIII (Fig. 5H) slightly prominent at the middle of posterior margin; tergite X (Fig. 5I) slightly emarginated at posterior margin; sclerotized spermatheca as in Figs 5J, 5K.

#### Distribution.

China (Guangxi Province: Huaping Nature Reserve).

#### Biological notes.

All the specimens were collected by shifting the mixture of bamboo leaves and broad tree leaves in dense forest.

#### Diagnoses.

In general facies, the new species resembles *Stenus zhaiyanbini* Tang & Li, 2012, but it may be distinguished by sparser and larger punctation of head, generally smaller elytral spots and the different sexual characters.

#### Etymology.

The specific name is derived from “Huaping”, the type locality of this species.

### Stenus zhujianqingi

Tang, Li & Huang sp. n.

urn:lsid:zoobank.org:act:F6AE9DCA-B411-44C1-A181-E3B8DABC24E4

http://species-id.net/wiki/Stenus_zhujianqingi

Figs 2 , 6

#### Type material.

**Holotype** ♂:“China: Zhejiang Prov., Lin’an City, Qingliangfeng N. R., 1750m, 9.VIII.2011, Zhu Jian-Qing leg.” “Holotype / *Stenus zhujianqingi* / Tang & Li” [red handwritten label] (SHNU). **Paratypes.** 13♂♂, 2♀♀, same data as for the holotype (1 pair in cPut, rest in SHNU); 4♂♂, 1♀, Qingliangfeng N. R., Longtangshan, 1100m, 12.V.2012, Chen, Ma & Zhao leg. (SHNU).

#### Description.

Brachypterous; body entirely black, anterior margin of labrum brownish, maxillary palpi yellowish brown, antennae and legs reddish brown.

BL: 4.0–4.3 mm; FL: 2.0–2.2 mm.

HW: 0.87–0.98 mm, PL: 0.71–0.84 mm, PW: 0.63–0.71 mm, EL: 0.74–0.82 mm, EW: 0.77–0.86 mm, SL: 0.53–0.64 mm.

Head 1.13–1.18 times as wide as elytra; interocular area with deep longitudinal furrows, median portion convex, not quite extending to level of inner eye margins; punctures round, mostly well delimited, slightly larger and sparser on median area than those near inner margins of eyes, diameter of large punctures about as wide as apical cross section of antennal segment II; interstices smooth, mostly much smaller than half the diameter of punctures. Antennae, when reflexed, not quite reaching posterior margin of pronotum; relative length of antennal segments from base to apex as 10.5: 7.5: 17.5: 10.5: 10: 7: 6.5: 5: 5: 5.5: 7.5. Paraglossa oval.

Pronotum 1.09–1.29 times as long as wide; disc with shallow short median longitudinal furrow, two indistinct impressions in anterior half, indistinct transverse impression in the middle, and two indistinct impressions in posterior half; punctures smaller than those of head, rugose and confluent; interstices smooth, slightly broader than diameter of punctures.

Elytra 0.95–1.00 times as long as wide, lateral margins with slight concavity before the middle; disc uneven with shallow longitudinal humeral impression, shallow sutural impression and shallow postero-lateral impression; suture convex; punctation and interstices similar to those of pronotum, but more rugose and confluent.

Hind tarsi 0.72 times as long as hind tibiae, tarsomeres IV distinctly bilobed.

Abdomen cylindrical; segments III–VI with tergites and sternites completely fused, without paratergites or sutures; tergite VII with palisade fringe; punctures on abdominal tergites III–VIII round to elliptic, gradually becoming smaller posteriad, punctures of tergite III large, of similar size as those of head; interstices smooth, mostly smaller than half the diameter of punctures.

Male. Sternite VII with posteromedian portion slightly flattened; sternite VIII (Fig. 6A) with shallow emargination at middle of posterior margin; sternite IX (Fig. 6B) with very long apico-lateral projections, posterior margin serrate; tergite X (Fig. 6C) with posterior margin slightly emarginated. Aedeagus (Figs 6D, 6E) with minute setae at sclerotized apex of median lobe; expulsion hooks (Fig. 6G) very large; parameres extending distinctly beyond apex of median lobe, a little folded at apical third, with about 17 setae on inner side (Fig. 6F).

Female. Abdomen broader than in male; sternite VIII (Fig. 6H) inconspicuously prominent at middle of posterior margin; tergite X (Fig. 6I) broader than in male; sclerotized spermatheca as in Figs 6J, 6K.

#### Distribution.

China (Zhejiang Province: Qingliangfeng N. R.).

#### Biological notes.

All the specimens were collected by shifting leaves in coniferous and broad-leaved mixed forest.

#### Diagnoses.

This species resembles *Stenus paradecens* Tang & Li, 2005 and *Stenus guniujiangensis* Tang & Li, 2005, but can be distinguished from both species by the rugose and confluent punctation of the pronotum and elytra. In addition, it also differs from *Stenus paradecens* by larger size and from *Stenus guniujiangensis* by longer elytra (see measurements in the modified key).

#### Etymology.

This species is named in honor of Mr. Jian-Qing Zhu, who collected most of the specimens of the new species.

To accommodate the new species, the recently published key to the Chinese species of the *Stenus indubius* group (Tang and Li 2012) is modified at couplets 2 and 5 as follows:

**Table:** 

2	Elytra shorter (EL/EW =0.86–0.93). Habitus: Fig. 4; sexual characters: Fig. 8. BL: 4.3–4.8 mm	*Stenus guniujiangensis* Tang & Li. China: Anhui
–	elytra longer (EL/EW = 0.95–1.01)	2a
2a	Body size larger (BL: 4.0–4.3 mm), punctation of pronotum and elytra rugose and confluent. Habitus: Fig. 2; sexual characters: Fig. 6	*Stenus zhujianqingi* sp. n. China: Zhejiang
–	Body size smaller (BL: 3.3–3.5 mm), punctation of pronotum and elytra well delimited. Habitus: Fig. 3; sexual characters: Fig. 7	*Stenus paradecens* Tang & Li. China: Anhui
5	Elytral marks larger, ranging from 3/5 to 4/5 as long and 1/2 to 2/3 as broad as the respective elytron. Habitus: Figs 7, 8 in Tang and Li (2012); sexual characters: Figs 40–50 in Tang and Li (2012). BL: 4.3–4.7 mm	*Stenus yinziweii* Tang & Li. China: Guizhou
–	Elytral marks smaller, ranging from 1/3 to 1/2 as long and 1/3 to 2/5 as broad as the respective elytron…5a
5a	Elytral marks on average smaller, about 1/3 as long and 1/3 as broad as the respective elytron; punctation of head sparser and coarser. Habitus: Fig. 1; sexual characters: Fig. 5. BL: 4.1–4.9 mm	*Stenus huapingensis* sp. n. China: Guangxi
–	Elytral marks on average larger, ranging from 1/3 to 1/2 as long and 1/3 to 2/5 as broad as the respective elytron; punctation of head denser and finer. Habitus: Figs 9, 10 in Tang and Li (2012); sexual characters: Figs 51–61 in Tang and Li (2012). BL: 4.2–5.1 mm	*Stenus zhaiyanbini* Tang & Li. China: Guizhou

**Figure 1.**
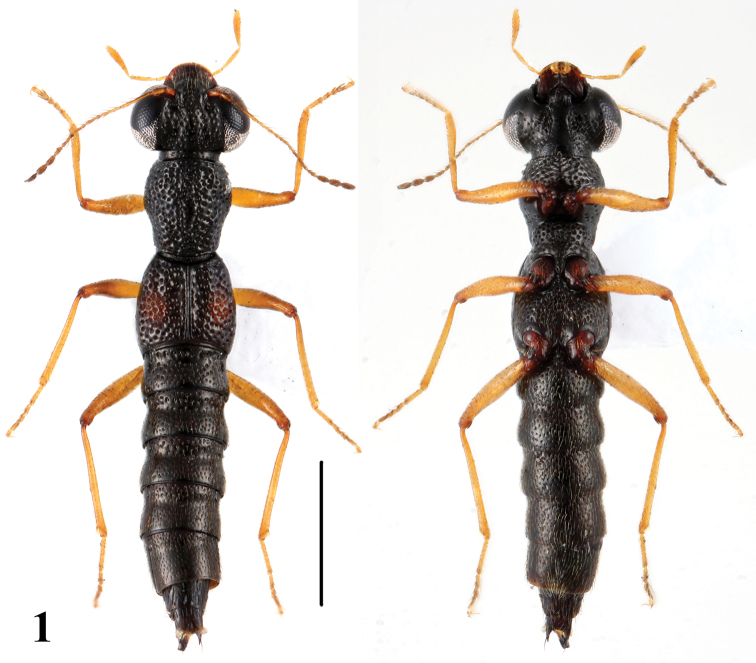
Habitus of *Stenus huapingensis* in dorsal and ventral view. Scale = 1 mm.

**Figure 2.**
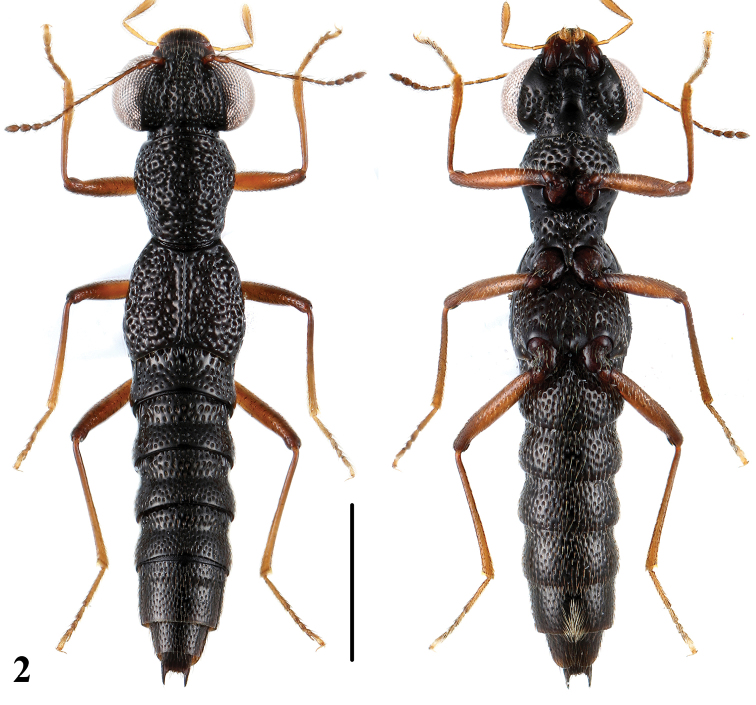
Habitus of *Stenus zhujianqingi* in dorsal and ventral view. Scale = 1 mm.

**Figure 3.**
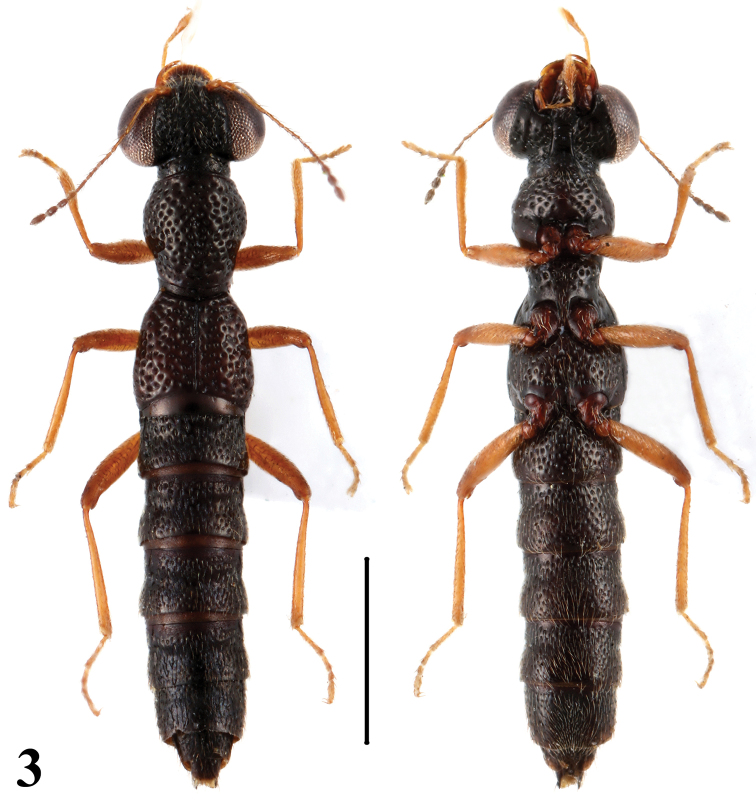
Habitus of *Stenus paradecens* in dorsal and ventral view. Scale = 1 mm.

**Figure 4.**
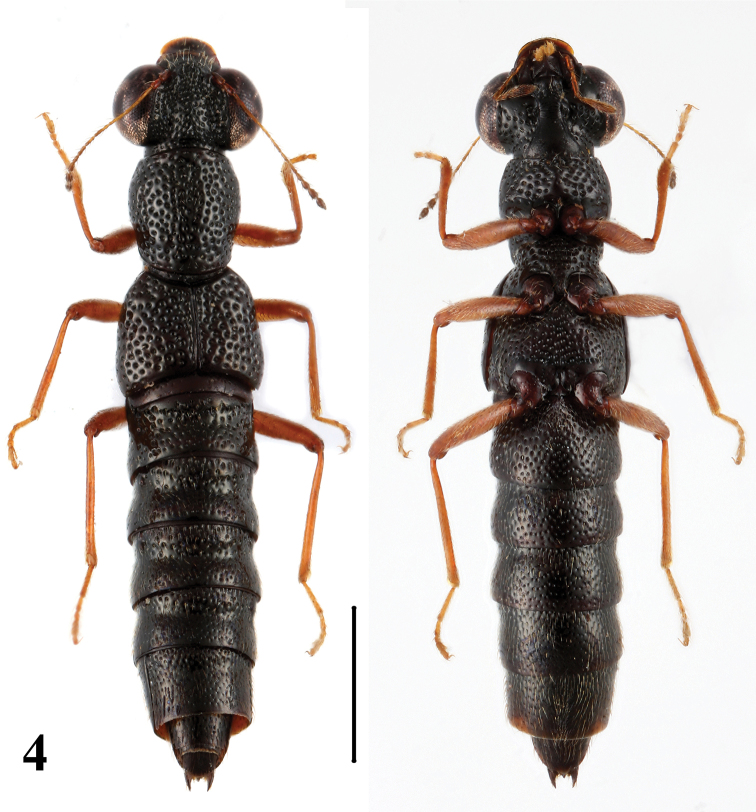
Habitus of *Stenus guniujiangense* in dorsal and ventral view. Scale = 1 mm.

**Figures 5.**
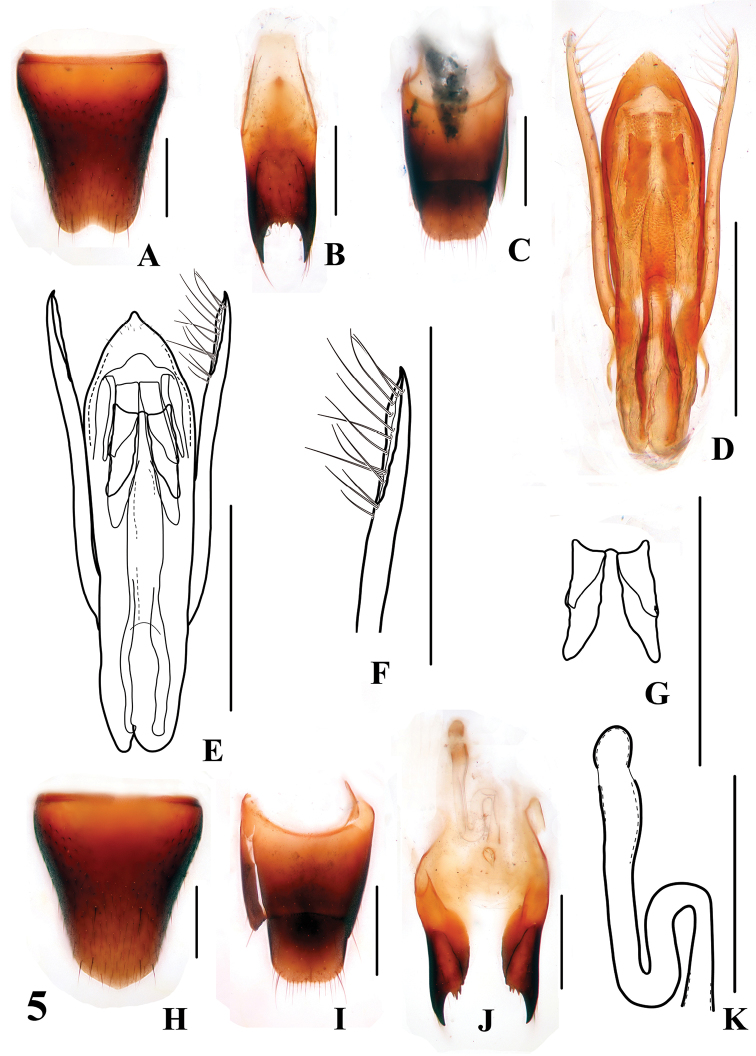
*Stenus huapingensis* **A** male sternite VIII **B** male sternite IX **C** male tergites IX, X **D, E** aedeagus **F** apical portion of paramere **G** expulsion hooks **H** female sternite VIII **I** female tergites IX, X **J** valvifers and spermatheca **K** spermatheca. Scales = 0.25 mm.

**Figures 6.**
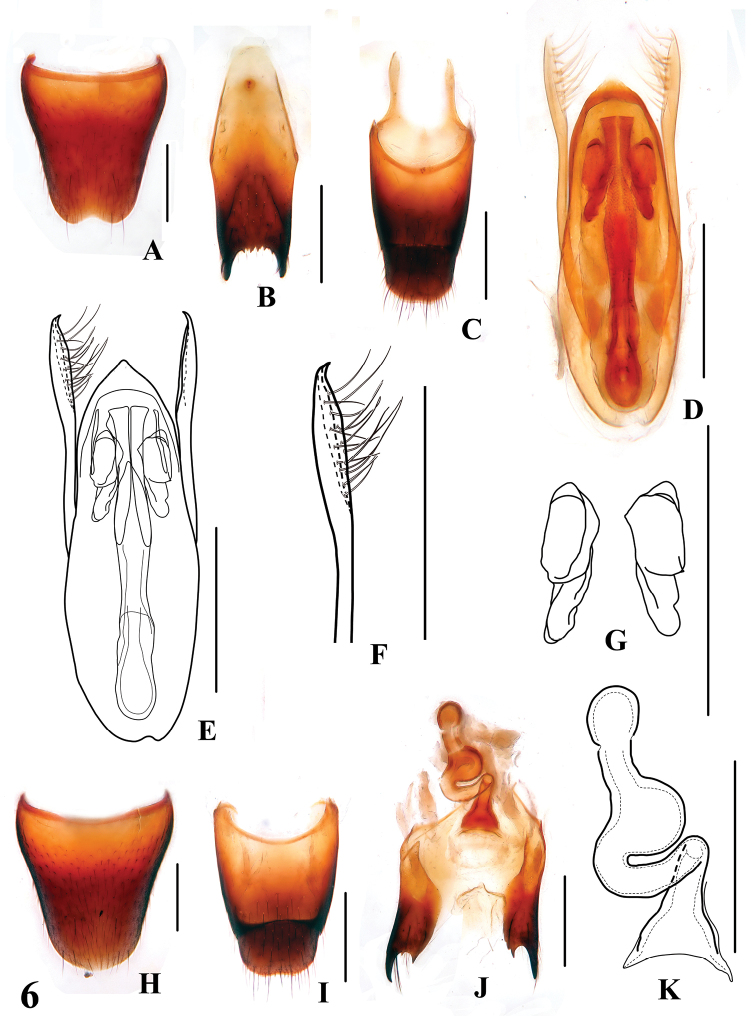
*Stenus zhujianqingi* **A** male sternite VIII **B** male sternite IX **C** male tergites IX, X **D, E** aedeagus **F** apical portion of paramere **G** expulsion hooks **H** female sternite VIII **I** female tergites IX, X **J** valvifers and spermatheca **K** spermatheca. Scales = 0.25 mm.

**Figures 7.**
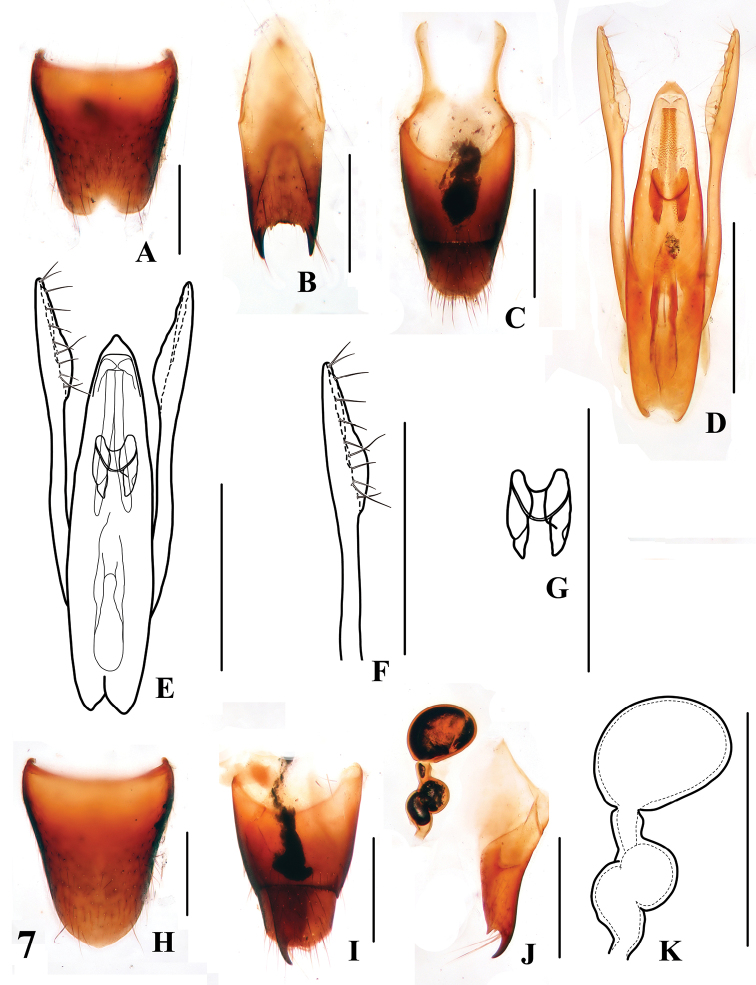
*Stenus paradecens* **A** male sternite VIII **B** male sternite IX **C** male tergites IX, X **D, E** aedeagus **F** apical portion of paramere **G** expulsion hooks **H** female sternite VIII **I** female tergites IX, X **J** valvifers and spermatheca **K** spermatheca. Scales = 0.25 mm.

**Figures 8.**
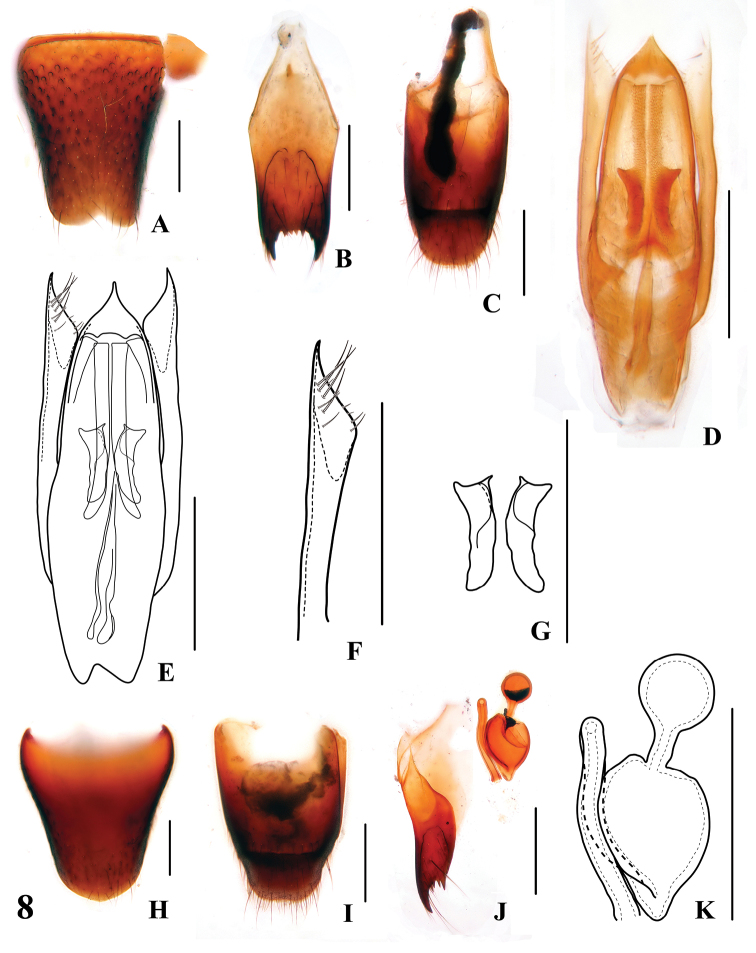
*Stenus guniujiangense* **A** male sternite VIII **B** male sternite IX **C** male tergites IX, X **D, E** aedeagus **F** apical portion of paramere **G** expulsion hooks **H** female sternite VIII **I** female tergites IX, X **J** valvifers and spermatheca **K** spermatheca. Scales = 0.25 mm.

## Supplementary Material

XML Treatment for Stenus huapingensis

XML Treatment for Stenus zhujianqingi
